# Use of left ventricle blood pool oxygenation-sensitive signal intensity as a measure of arterial hemoglobin saturation

**DOI:** 10.1186/1532-429X-18-S1-P52

**Published:** 2016-01-27

**Authors:** Kai Homer, Kady Fischer, Dominik P Guensch, Nancy Shie, Julie Lebel, François Roubille, Matthias G Friedrich

**Affiliations:** 1 Philippa & Marvin Carsley CMR Centre, Montreal Heart Institute, Montreal, QC Canada; 2 Anaesthesiology and Pain Therapy, Bern University Hospital, Bern, Switzerland; 3 Cardiology and Radiology, Université de Montréal, Montreal, QC Canada; 4 Department of Cardiology, University Hospital of Montpellier, Montpellier, France

## Background

Oxygenation-sensitive cardiovascular magnetic resonance (OS-CMR) relies on the attenuating effects of deoxyhemoglobin as an intrinsic contrast. When using OS-CMR to assess myocardial oxygenation, failing to account for effects on myocardial oxygen-sensitive signal intensity (OS-SI) due to arterial hemoglobin desaturation could confound results attributed to changes in perfusion and tissue metabolism. In particular, this can be relevant when using breath-holds as a vasodilating mechanism. Using an animal model we first assessed if arterial saturation can be non-invasively measured using the blood pool signal (SI) in OS-CMR images, and secondly if this effect is relevant for human studies.

## Methods

In eight anaesthetized healthy swine, cannulas were placed in the femoral artery for blood sampling. Arterial and venous blood samples were taken from a human cohort of 36 healthy humans and 29 obstructive sleep apnea syndrome (OSAS) patients. All subjects performed a breathing maneuver of 60s hyperventilation followed by a long end-expiration breath-hold. In humans, the breath-hold duration was voluntary and analyzed for 30s, and in animals, manual ventilation performed and paused for 90s. OS-CMR images were acquired continuously throughout the breath-hold in a mid-slice short-axis slice with a bSSFP sequence (FA=35°, TR=3.4 ms) at 3T. SI values were captured in the left ventricle blood pool at end systole and expressed as a change over the breath-hold.

## Results

In healthy swine, blood pool SI decreases by -14.3% ± 6.5% (*p*<0.01) over the course of the breath hold. This decrease correlates significantly (r = 0.80, *p*<0.05) with arterial hemoglobin saturation. In healthy and OSAS patients, the average SI of the group does not decrease significantly over the course of the breath hold. However, at 30 seconds of apnea, 10.7% (*n*=3) of OSAS patients' SI% values were lower than one standard deviation below the healthy group mean SI% of -1.4% ± 5.6%.

## Conclusions

In a fundamental animal study, changes in blood pool SI after breathing maneuvers parallel changes in arterial hemoglobin saturation. Thus, SI may serve as a baseline measure of arterial hemoglobin saturation when assessing myocardial oxygenation using OS-CMR. In some patients, a significant desaturation of the blood could act as a confounding variable in myocardial OS-SI measurements.

**Figure 1:**
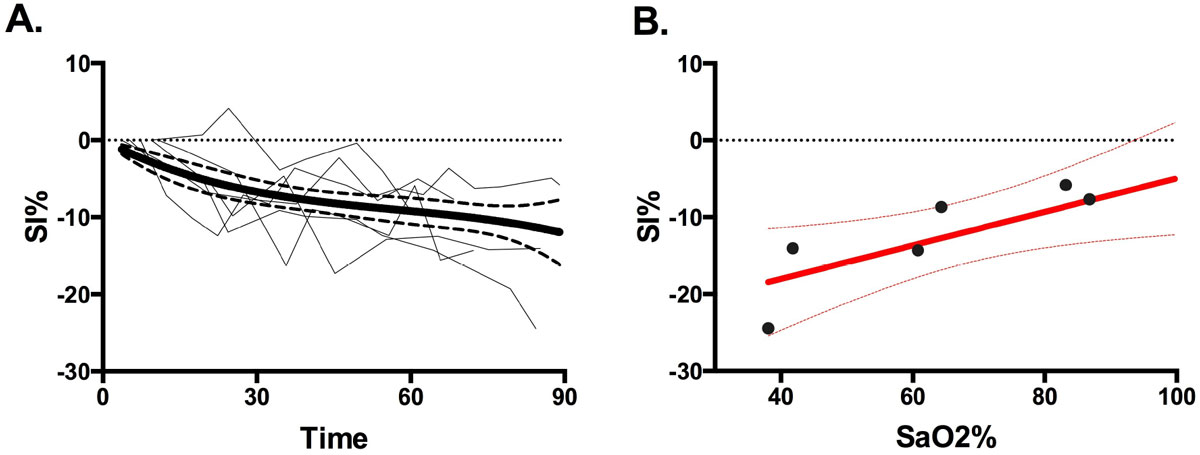
**Change in bloodpool signal intensity (SI) in swine decreases throughout a forced pause in ventilation (A), correlating (r = 0.80, p < 0.05) to the drop in arterial hemoglobin saturation (B)**.

